# Iron Chelation as a Potential Therapeutic Approach in Acute Lung Injury

**DOI:** 10.3390/life13081659

**Published:** 2023-07-30

**Authors:** Xiyang Zhang, Juan Zhou, Bruce E. Holbein, Christian Lehmann

**Affiliations:** 1 Department of Anesthesia, Pain Management and Perioperative Medicine, Dalhousie University, Halifax, NS B3H 1X5, Canada; zhangxiyang17@gmail.com (X.Z.); juan.zhou@dal.ca (J.Z.); 2 Department of Anesthesiology, Nanfang Hospital, Southern Medical University, 510515 Guangzhou, China; 3 Department of Microbiology & Immunology, Dalhousie University, Halifax, NS B3H 1X5, Canada; beholbein@sympatico.ca; 4 Department of Physiology & Biophysics, Dalhousie University, Halifax, NS B3H 1X5, Canada; 5 Department of Pharmacology, Dalhousie University, Halifax, NS B3H 4R2, Canada

**Keywords:** acute lung injury, iron overload, ROS, ferroptosis, iron chelator

## Abstract

Acute lung injury (ALI) has been challenging health care systems since before the COVID-19 pandemic due to its morbidity, mortality, and length of hospital stay. In view of the complex pathogenesis of ALI, effective strategies for its prevention and treatment are still lacking. A growing body of evidence suggests that iron dysregulation is a common characteristic in many subtypes of ALI. On the one hand, iron is needed to produce reactive oxygen species (ROS) as part of the immune response to an infection; on the other hand, iron can accelerate the occurrence of ferroptosis and extend host cell damage. Iron chelation represents a novel therapeutic strategy for alleviating lung injury and improving the survival of patients with ALI. This article reviews the current knowledge of iron homeostasis, the role of iron in ALI development, and potential therapeutic targets.

## 1. Introduction

Acute lung injury (ALI) represents the result of the pathophysiological response to a noxious trigger and is characterized by compromised pulmonary gas exchange. The causes of ALI can be direct (e.g., pneumonia, including SARS-CoV-2) or indirect (e.g., due to sepsis) [1]. Clinically, ALI can progress to acute respiratory distress syndrome (ARDS), graded on the degree of hypoxemia, with high morbidity and mortality, which remains up to 40% [2,3]. Previous studies have shown that the pathogenesis of ALI/ARDS involves oxidative stress, apoptosis, and other pathways of inflammation [4,5,6]. Recent studies have demonstrated that iron dysregulation also plays an important role in the pathogenesis of ALI/ARDS [7].

Indeed, through targeting the molecular and metabolic pathways that regulate cellular defense against infection and cytotoxicity, iron and iron-related proteins have been linked to the development of pulmonary disease. Alveoli are composed of alveolar macrophages and types 1 and 2 alveolar epithelial cells, and all of these three major cell types are active in the maintenance of lung iron homeostasis [8]. For example, it is reported that LPS-stimulated mouse alveolar macrophages increased the expression of hepcidin mRNA and decreased transmembrane protein ferroportin (FPN) mRNA, leading to a reduction in iron efflux, and the results suggest that the lung hepcidin–FPN axis may correlate with iron homeostasis during alveolar macrophage injury induced by LPS [9]. Recently, long non-coding RNAs (lncRNAs) have been considered as important mediators of iron metabolism [10]. Through regulating the amount and function of iron, seven iron metabolism–related lncRNAs are recognized as an independent prognostic factor for the overall survival of lung adenocarcinoma patients. Therefore, iron metabolism and its regulatory mechanism may serve as a new diagnostic and therapeutic target in lung cancer [11]. In addition, during the pathophysiological process of infection caused by various bacteria or viruses, lactoferrin (Lfn) can rebalance the systemic iron concentrations, and impact the host immune response, so as to modulate the inflammation response and the promotion of antiviral gene expression [12,13,14].

However, the specific regulation of iron and iron-related proteins in response to lung injury or infection is still poorly understood. The purpose of this review is to explore recent scientific advances in understanding the role of iron regulation in ALI/ARDS.

## 2. Iron in Health and Disease

### 2.1. Importance of Iron in Human Health

Iron is an essential trace mineral for normal biological function in almost all organisms. In order to maintain baseline homeostasis and resupply the minor unregulated loss of iron, 25 mg/day of iron is required for a human adult. About 65% of the total iron in humans is stored in RBCs in the form of hemoglobin, 20% is allocated in macrophages and hepatocytes, 10% is distributed in muscle fibers as myoglobin, and 5% is in bone marrow [15].

**Figure 1:**
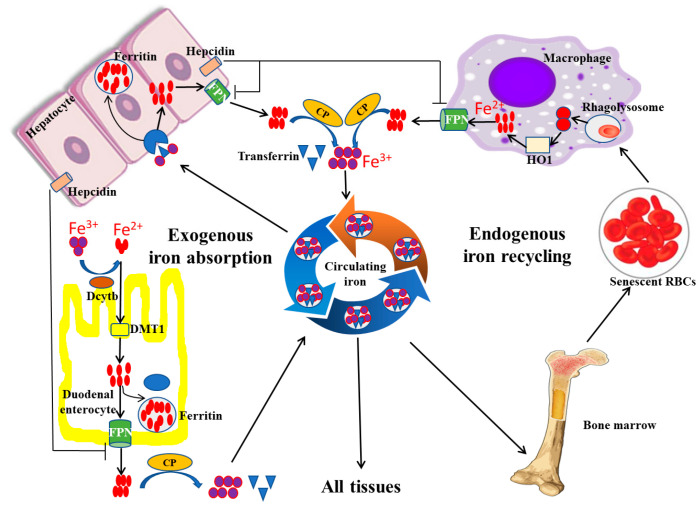
Iron absorption, recycling, and storage under physiological conditions. Exogenous iron absorption and endogenous iron recycling are two sources of iron in the human body. During the process of iron absorption and recycling, DcytB and DMT1 are responsible for the uptake of Fe^2+^ from the lumen, and macrophages are in charge of the cyclic utilization of iron from senescent erythrocytes. In addition, excess iron is usually stored in the hepatocyte in the form of ferritin, and FPN can export Fe^2+^ into the blood. FPN, ferroportin; HO-1, heme oxygenase 1; CP, ceruloplasmin; DcytB, duodenal cytochrome B; DMT1, divalent metal transporter 1 (modified according to Abbasi et al., 2021 [18]).

Under physiological conditions, there are two main sources of iron absorption in the body, namely endogenous iron absorption and exogenous iron absorption (Figure 1). Endogenous absorption is mainly from the recycling of iron from senescent erythrocytes. In the process of clearance of aging or damaged erythrocytes, macrophages phagocytose the erythrocytes and decompose heme, a component of hemoglobin with iron bonded, using heme oxygenase 1 (HO-1) to release iron, which can be recycled back into circulation by the iron exporter ferroportin (FPN), a transmembrane protein on the cell surface (Figure 1). Exogenous iron absorption mainly refers to dietary iron. Generally, about 1~2 mg of iron, in the form of either heme iron (Fe^2+^) or non-heme iron (Fe^3+^), is absorbed daily by duodenal enterocytes through different mechanisms (Figure 1). Fe^2+^ is directly absorbed in the apical surface of enterocytes via iron transport protein, divalent metal transporter 1 (DMT1), whereas Fe^3+^ is not bioavailable and needs to be converted to Fe^2+^ by duodenal cytochrome B (DcytB), a converting enzyme, before being absorbed by the apical membrane of duodenal enterocytes via DMT1 [16]. Absorbed iron in duodenal enterocytes can be transported through FPN into the circulation to play its physiological role [17].

To maintain iron homeostasis, hepcidin, a peptide hormone secreted by the liver, plays an important role by regulating the expression of FPN on the cell surface. Hepcidin can reduce FPN expression by binding FPN and inducing its internalization and degradation in lysosomes. In physical conditions, the absorbed iron can be stored and iron can be transported into the bloodstream via FPN. When iron levels exceed the body’s requirements, the increased secretion of hepcidin inhibits FPN expression, resulting in reduced iron transportation from cells into the blood circulation [19].

Transferrin (Tf), an iron transport protein, is responsible for the transportation and distribution of almost all iron within circulating blood (Figure 1). Specifically, most of the iron in the bloodstream exists in the form of Fe^3+^. About 25~30 mg/day of iron can be delivered by Tf from plasma to developing erythroid cells in the bone marrow for heme biosynthesis. Lower amounts of iron, up to 5 mg/day, are supplied to other tissues via Tf in order to meet various physiological needs. The pathway for iron transportation from Tf into cells is not fully defined, but the current evidence suggests that it involves endocytosis of the transferrin receptor (TfR) complex; the binding force between Tf and Fe^3+^ is then reduced, so as to allow for the release of iron from transferrin [20]. As a result, the free iron can then move into the cytoplasm and be used by the cell.

Under normal conditions, iron above immediate cell needs is safely stored within ferritin. Ferritin, as a hollow globular protein, is usually composed of 24 H and L subunits, so it can store up to 4500 ferrous ions. Furthermore, because hepatocytes are the major site for ferritin synthesis, the liver is the primary iron storage organ. In addition, some recycled iron is reincorporated into the bone marrow. When the iron demand increases, the stored iron in both the liver and bone marrow is mobilized to increase the iron content in circulation.

Serving as a prosthetic group for a variety of proteins, iron plays an important role in various vital physiological processes of cells. For example, iron is required for cell viability and proliferation. Iron functions as a catalyst for enzymes and plays a key role in DNA synthesis and repair, and cellular energy metabolism. Moreover, iron is also thought to play a role in the immune system. However, iron overload can attenuate the phagocytosis of macrophages, and affect the function of T lymphocytes, so as to disrupt the immune response [21,22].

By acquiring iron from hosts, invading microbes can also utilize iron to promote their own growth, disturb the homeostatic balance of the body, and bring potential adverse consequences [23,24,25]. Therefore, it is a physiological challenge to maintain the balance of the body’s requirement for iron, particularly in states of infection and inflammation.

### 2.2. Iron Regulation

In order to maintain an appropriate iron balance, organisms have evolved complex systemic homeostatic and cellular iron transport mechanisms. For example, hepcidin, produced primarily by hepatocytes, is the key regulator of systemic iron homeostasis. When the iron circulating concentration is increased in plasma, the body responds by elevating hepcidin release, which can downregulate FPN mRNA and protein levels, reducing the efficiency of delivering iron from storage sites to plasma, and thus lowering the plasma iron level [19,26]. Similarly, when the iron concentration is decreased, the body responds by reducing hepcidin release, which can upregulate the expression levels of FPN, partly resulting in a recovery of iron reabsorption and an increase in iron concentration [22].

In addition to the hepcidin/FPN pathway, the iron regulatory protein/iron-responsive elements (IRP/IRE) system also plays a key role in maintaining iron homeostasis. IREs are mainly located in the untranslated regions of mRNAs encoding proteins related to the absorption, storage, utilization, and export of iron. Interestingly, the 5′-end IREs mainly regulate gene expression related to iron storage and transport, such as ferritin and FPN. However, the 3′-end IREs are closely related to the expression of iron absorption genes, such as TfR1 and DMT1. Moreover, IRP1 and IRP2, via binding to IREs of the 5′ and 3′ regions, can control the mRNA translation or stability. Generally, the translation of the mRNAs, including ferritin and FPN, will be inhibited under the condition of IRPs combined with 5′ IREs, whereas the stability of mRNAs, including TfR1 and DMT1, will be reinforced when IRP binds to 3′ IREs. For example, under iron overload conditions, IRP1 is converted to c-aconitase by SCF (SKP1-CUL1-F-box) E3 ligase complex and IRP2 is degraded by proteasomes, resulting in decreased IRP binding to IREs. This dissociation of IRP–IRE interaction will increase the translation of FPN and ferritin mRNA and disturb TfR1 and DMT1 mRNA, resulting in an increase in iron storage and export and a decline in iron absorption [8,27,28,29].

**Figure 2:**
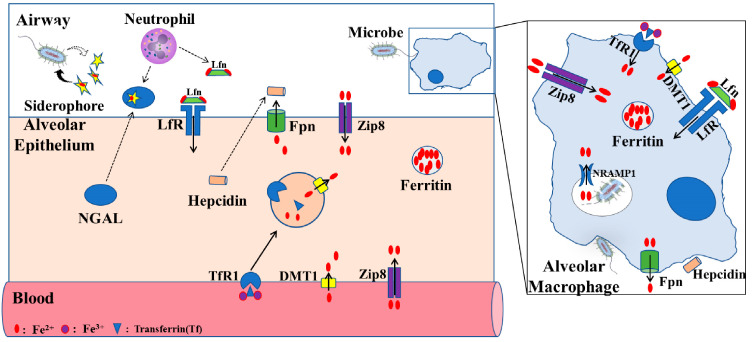
Proposed iron regulation in lung. The level of iron in alveolar epithelial cells is mainly regulated by TfR1, DMT1, ZIP8, LfR, and FPN receptors. Alveolar macrophages, through phagocytosis of bacteria, play an important role in lung defense response. Fpn, ferroportin; ZIP8, zinc transporter SLC39A8; Lfn, lactoferrin; LfR, lactoferrin receptor; DMT1, divalent metal transporter 1; TfR1, transferrin receptor 1; NRAMP1, natural resistance–associated macrophage protein 1NGAL, neutrophil gelatinase–associated lipocalin (modified according to Zhang et al., 2019 [35]).

Recent studies in mouse models have begun characterizing the roles of zinc transporter SLC39A8 (ZIP8) and other soluble proteins in regulating body iron homeostasis. ZIP8 is a zinc transporter and is encoded by the SLC39A8 gene, and has also been proven to have the ability to import iron into the cytosolic space [30]. In in vitro and animal studies, ZIP8 expression can be greatly increased after inflammatory stimulation with LPS, suggesting that increased ZIP8 via iron transporting has a function in host defense [31,32] (Figure 2). Lipocalin-2 is a soluble protein and is secreted by epithelial cells and neutrophils, and can also promote cellular and tissue iron uptake through the siderophore–lipocalin-2 complex. It is reported that circulating concentrations of lipocalin-2 are positively associated with adiposity, hypertriglyceridemia, hyperglycemia, insulin resistance, and circulating levels of C-reactive protein. Moreover, lipocalin-2 can bind to iron with a much higher affinity than the endogenous carrier protein transferrin, thus potentially aiding pathogen survival and growth during infection [25,33,34].

### 2.3. Iron in Pathology and Diseases

Iron deficiency results in the impairment of multiple cellular functions and, in particular, erythropoiesis and red blood cell heme contents are reduced [18,36]. Research has demonstrated that iron deficiency is associated with up to 60% of patients with cardiovascular disease, including coronary artery disease, heart failure, and pulmonary hypertension. In elderly patients with reduced ejection fraction, both anemia and iron deficiency may confer independent risk due to a poor prognosis [37]. Iron deficiency is usually caused by an underlying inflammatory disease, such as inflammatory bowel disease (IBD) or chronic kidney disease (CKD) [38]. In IBD, iron deficiency is markedly associated with decreased intestinal iron absorption, malnutrition, chronic inflammation, and blood loss. In patients diagnosed with CKD, anemia is induced by disturbed renal erythropoietin (EPO) production, which is responsible for erythropoiesis. Iron supplementation or recombinant EPO have been used to improve anemia in these patients [39]. In addition, iron deficiency is closely related to tumor progression. At the cellular level, the expression of iron metabolism–related proteins is altered in cancer. For example, TfR1 and DMT1, which increase iron uptake, are highly overexpressed in many tumor types, increasing intracellular iron levels [40,41]. Also, it is reported that FPN, which is in charge of iron release, is downregulated in prostate and breast cancer [42,43]. Therefore, iron deficiency, especially iron-deficiency anemia, has become one of the most important contributors to the global burden of disease, which mainly affects children, as well as people in low-income countries [44].

On the other hand, iron overload also has adverse consequences for the host, including oxidative stress, vascular dysfunction, ferroptosis, and peroxidation of lipid membranes [45,46,47]. Generally, under pathological conditions, iron overload mainly refers to non-transferrin-bound iron. Because humans lack a proper physiological pathway to clear out iron, it is important for iron to be maintained in homeostasis via a complex feedback mechanism between iron uptake, utilization, and storage. However, under pathological conditions, this balance will be disturbed. Initially, the excess iron can be safely combined with transferrin or stored in ferritin. However, transferrin will be saturated quickly by an accumulation of iron, raising the level of non-transferrin-bound-iron, which is rapidly taken up by various organs. As a result, the body will have a pathological situation of iron overload, resulting in organ damage. For example, a clinical study showed that due to high glucose toxicity in diabetic patients, the release of serum iron was inhibited, which resulted in iron overload. What is more, by facilitating lipid peroxidation and catalyzing excess hydroxyl free radicals, the increase of serum iron can promote the occurrence of acute kidney injury (AKI); therefore, iron overload is considered an independent risk factor for AKI in critically ill patients with diabetes [48]. It is also reported that iron is necessary for the continuous and rapid proliferation of cancer cells, resulting in accelerated cancer cell growth and increased morbidity and mortality in cancer patients [22]. Moreover, excess iron can promote the generation of free radicals and reactive oxygen species, which damage healthy cells and induce inflammation.

Furthermore, increased iron availability is also associated with the increased virulence of multiple microbial pathogens [49,50]. Toni et al. described the link between iron overload and an increased susceptibility to various invasive fungal infections, and suggested that reducing the level of iron may be a better way to prevent infections in patients with hematological malignancies [51]. Although different bacteria and viruses have different ways of acquiring and utilizing iron, they all use iron with the same goal of promoting their own growth, increasing virulence, and ultimately increasing pathogenicity. Therefore, the use of iron chelators to limit iron availability and control microbial survival may be a useful way to hinder the development of infections [52].

## 3. Iron and ALI

### 3.1. Direct ALI

**Figure 3:**
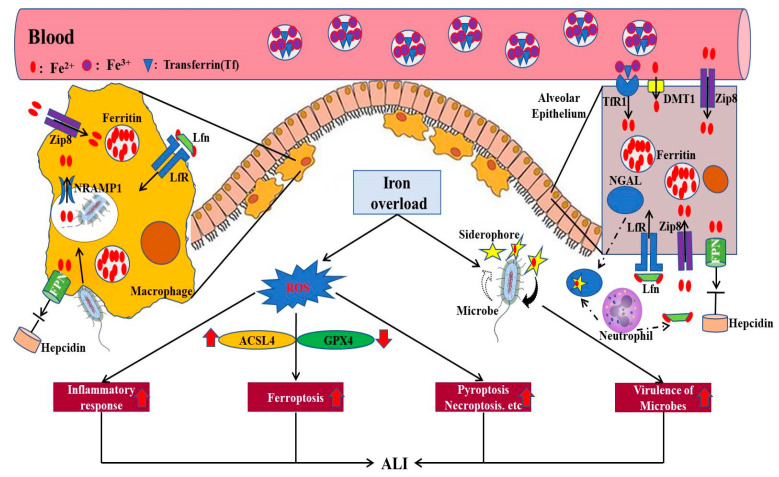
The detrimental effects of iron overload in the development of ALI. Overloaded iron in the lung, including alveolar epithelium and macrophages, can accelerate the production of ROS, thus inducing the inflammatory response, ferroptosis, pyroptosis, and necroptosis of pulmonary epithelial cells. In addition, excess iron can promote the virulence of microbes aggravating the development of ALI. FPN, ferroportin; NRAMP1, natural resistance–associated macrophage protein 1; ZIP8, zinc transporter SLC39A8; Lfn, lactoferrin; LfR, lactoferrin receptor; DMT1, divalent metal transporter 1; TfR1, transferrin receptor 1; NGAL, neutrophil gelatinase–associated lipocalin; ROS, reactive oxygen species; GPX4, glutathione peroxidase 4; ACSL4, Acyl-CoA synthetase long-chain family 4 (modified according to Zhang et al., 2019 [35]).

ALI can occur as a result of a direct pathogenic insult to the lung, such as pneumonia induced by viruses or bacteria. Recently, the global pandemic COVID-19, caused by severe acute respiratory syndrome coronavirus 2 (SARS-CoV-2), presented clinically with severe ARDS [53,54,55]. The pathogenesis of COVID-19 includes inflammation, hypercoagulation, and immune dysfunction, and is associated with iron dysregulation [56]. First, SARS-CoV-2 might have a direct effect on hepcidin regulation [57]. SARS-CoV-2 spike protein has similarities to hepcidin, which potentially has an impact on ferroportin activity [58]. Moreover, increased intracellular iron during SARS-CoV-2 infection can induce iron-dependent peroxidation, leading to cellular apoptosis and ferroptosis [59]. It has been reported that hepcidin dysregulation and hyperferritinemia are significantly associated with multiple organ failure in COVID-19 patients [60]. Second, lipid damage catalyzed by iron overload can exert a direct causative effect on ferroptosis, which is more likely to amplify cell death and trigger a series of other inflammatory-related immune regulatory responses [61] (Figure 3).

There is also an abundance of evidence that iron is essential for various bacterial physiological and metabolic processes, and reducing the level of iron may have a positive effect on outcomes in ALI/ARDS [62]. For example, it has been demonstrated that macrophage ferroportin is significantly increased in ALI/ARDS patients with bacterial pneumonia [63]. In turn, the degradation of ferroportin in macrophages not only limits the availability of iron to bacteria, but also promotes tissue restoration, eventually attenuating lung injury induced by pneumonia. In addition, iron overload is associated with the increased virulence of multiple microbes, including *Yersinia enterocolitica*, *Escherichia coli,* and *Klebsiella pneumonia* [24,64,65]. During infection, the body uses some iron-binding proteins to control the iron supply accessible to microbes, such as neutrophil gelatinase–associated lipocalin (NGAL or lipocalin-2), lactoferrin, and natural resistance–associated macrophage protein 1 (NRAMP1). In LPS-treated mice, lipocalin-2 knockout significantly decreased iron-stained macrophages and oxidative stress, suggesting that lipocalin-2 is a potential therapeutic target for LPS-induced acute lung injury [62]. It has also been confirmed that levels of lactoferrin correlate with the severity of infectious pneumonia and sepsis induced by bacteria and viruses [12,66]. NRAMP1, as a divalent metal transporter and a vital player in the host–pathogen battle for phagolysosomal iron, may have a broader role in regulating macrophage iron homeostasis through the hepcidin–Nramp1 axis [67] (Figure 3).

### 3.2. Indirect ALI

Indirect ALI can be caused by the systemic inflammatory response in conditions such as sepsis, trauma, ischemia reperfusion (I/R), and transfusion-related ALI [35]. For example, during lung I/R, massive amounts of free radicals and ROS can be released, and these harmful substances not only recruit pro-inflammatory leukocytes, but also disrupt the integrity of epithelial cells, resulting in alveolar damage and impaired lung function [68]. Via Fenton and Haber–Weiss reactions, ROS can be catalyzed by iron, so dysregulation of iron homeostasis may promote ROS generation, exacerbating the inflammatory response in indirect ALI [69]. In addition, the development of ALI, as examined in numerous basic and clinical studies, has shown strong correlations with iron and iron-related proteins. Dixon et al. described the different roles of iron in triggering cell death, suggesting that peroxides are easy to convert to damaging radicals with enhanced cytotoxicity in the presence of iron [70]. One clinical study demonstrated that pulmonary macrophages originating from ARDS patients were characterized by an increased expression of FPN [63]. In addition, excess iron and ferritin were also associated with the LPS-induced ALI and the early phases of silicosis [71,72]. Therefore, as an acute phase reactant, serum ferritin is believed to have definite value in predicting the occurrence and development of ALI/ARDS in two clinical studies, although there is no correlation of ferritin with the degree of hypoxia, time of invasive ventilation, or mortality [73,74]. However, the exact causative effects of iron and iron-related proteins on ALI/ARDS remain to be more fully elucidated.

In addition to well-known cell death pathways, such as necroptosis and apoptosis, an increasing number of studies have begun to investigate the role of ferroptosis, a newly understood type of cell death in various diseases, including ALI/ARDS [75]. Ferroptosis is an iron-dependent, non-apoptotic mode of cell death characterized by the accumulation of a large amount of iron and ROS, resulting in oxidative cell death. Execution of the ferroptosis-relevant pathological process relies on lipid peroxidation, which is regulated by both Acyl-CoA synthetase long-chain family 4 (ACSL4) and glutathione peroxidase 4 (GPX4) [68]. ACSL4, as one of the main enzymes in pro-ferroptotic lipid metabolism, can catalyze the synthesis of arachidonic acid (AA)-CoA, which promotes the formation of phospholipid hydroperoxide (PL-OOH), resulting in the development of classical ferroptosis [76]. On the contrary, GPX4 is a lipid repair enzyme having the capacity to inhibit lethal lipid peroxidation in phospholipid bilayers. Thus, cells with knockout or inactivation of GPX4 are more susceptible to ferroptosis [77,78,79] (Figure 2).

Evidence has shown that ferroptosis plays a key role in ALI induced by I/R or lipopolysaccharide (LPS)-mediated sepsis, and the mechanism may be related to the disruption of iron homeostasis [80,81,82]. For example, Xu et al. reported that lung I/R induced significant increases in iron content and accumulation of lipid peroxidation in lung tissues. What is more, through the Fenton and Haber–Weiss reactions, the increased iron can trigger the formation of abundant pro-ferroptotic ROS, along with key protein (GPX4 and ACSL4) expression alteration, boosting the ferroptotic damage after lung I/R [68]. It was also demonstrated that ferrostatin-1 (Fer-1), via reducing the total iron level, showed the ability to inhibit lipid peroxidation, so as to work against ferroptosis in the model of LPS-induced acute lung injury [83]. Zhang et al. have verified that Yes-associated protein 1 (YAP1) can alleviate sepsis-induced acute lung injury via inhibiting ferritinophagy-mediated ferroptosis [84]. Therefore, iron uptake mediated via transferrin receptors and iron-dependent radical formation are crucial prerequisites for the execution of ferroptosis [85].

## 4. Potential Therapeutics

Because of the importance of iron dysregulation in the development of ALI/ARDS, especially the deleterious effects of intracellular iron overload, potential therapeutics to manipulate iron availability to alleviate the toxic side effects of excess iron have become of interest. In order to treat iron overload, four possible approaches have been suggested to reduce the level of iron. First, conventional methods including dietary restriction, phlebotomy, and chelators are considered effective ways to reduce iron load in the body [86]. Second, modulating the specific iron transporters, such as hepcidin and FPN, might provide a potential therapeutic approach [87]. Third, the use of an iron competitor to interfere with iron uptake, such as cationic metal gallium, is another feasible approach to decrease the level of iron [88]. Fourth, due to the availability of novel chelation substances, local or systemic administration of iron chelators will play a more effective therapeutic role [35]. Among the approaches to treat iron overload, iron chelation is the most widely used clinically, and progress is continuously happening in this field [18].

To date, there are only three clinically approved iron chelators, including: deferoxamine (DFO), deferiprone (DFP), and deferasirox (DFX). Although the first use of iron chelators can be traced back to 1968, there are significant limitations that have hindered the clinical application of these iron chelators [18]. For example, due to its large hydrophilic structure, DFO can be only administered intravenously or subcutaneously, but not orally, and the half-life is 20 to 30 min [18]. Compared with DFO, DFP is a small-molecular-weight iron chelator with a longer half-life of 3 to 4 h, which also offers the advantage of its oral bioavailability. Moreover, DFX was approved by the FDA in 2005. DFX is also orally bioavailable, and has the longest half-life among the chelators (12–16 h). Due to its improved tolerability profile, DFX is the preferred chelator for administration in children.

Although there are limitations, DFP can significantly alleviate LPS-induced lung injury by inhibiting lipocalin-2 expression [62]. Some newer synthetic iron chelators with fewer adverse effects have also shown promising results in reducing iron-mediated toxicity, but these investigations are still largely in the preclinical or early clinical stage [18]. As a new copolymer chelator with a relatively low molecular weight (9 kDa), DIBI (poly [(*N*-vinylpyrrolidone)-co-(3-hydroxy-1-[*N*-(methacrylamido) ethyl]-2-methylpyridin-4(1H)one) has several advantages. For example, because iron can be tightly sequestered in the interior of the polymer, DIBI has a higher binding affinity for iron [89]. In addition, compared to conventional iron chelators, DIBI has a low toxicity profile and has been extensively studied for a wide variety of iron chelation applications [90,91,92,93,94]. Our group has recently reported that through binding to extracellular iron and reducing overall iron bioavailability, DIBI administration significantly attenuated LPS-induced tissue damage, inhibited inflammatory mediator release, and improved microcirculation in ALI [95]. Experimental studies utilizing iron chelators in ALI are shown in Table 1.

In addition to the iron chelators listed above, including DFO, DFP, DFX and DIBI, numerous small-molecule chelators have been studied for different iron-induced pathogenesis. For example, chelators of the quinoline family for the treatment of Alzheimer’s disease and Huntington’s disease are at the stage of in vitro experiments and phase II studies, respectively [106,107]. However, in order to possess maximal efficacy and minimal toxicity, the design of iron chelators requires an optimized half-life, accurate targetability for tissue, and a high affinity for iron, so some new challenges need to be solved in the future, through means such as (1) via improving selectivity toward iron (Fe^3+^) to promote iron chelation efficiency; (2) through reducing unwanted organ/tissue accumulation and increasing adequate excretion routes to lower the toxicity of biodegradable components. Interestingly, it is has been reported that the release of small-molecular drugs can be controlled by the utilization of polymer nanoparticles, which represents a promising approach to improve the organ-targeting efficiency of the chelators [108,109].

## 5. Conclusions

In summary, iron mobilization and disturbed iron homeostasis are significant pathophysiological characteristics in ALI/ARDS. On the one hand, iron promotes the release of ROS and thus aggravates the inflammatory response in the lung tissue, and, by activating lipid peroxidation, iron accelerates the occurrence of ferroptosis, which causes further cell damage. At the same time, iron can increase the activity of various pathogens and ultimately exacerbates the progression of ALI. Correspondingly, iron chelation therapy has been investigated for the experimental treatment of ALI, with promising results. Therefore, in order to design and develop new iron chelators with maximal efficacy and minimal toxicity, we still need to conduct extensive basic and clinical studies.

## Figures and Tables

**Table 1:** Iron chelation in experimental ALI.

Model	Iron Chelators	Readout	Outcome	Refs
RAW264.7 cells	DFP	Lipocalin-2	IL-6, TNF-α, iNOS ↓ Oxidative stress ↓	[62]
Mice	DIBI	Leukocyte adhesion Inflammatory mediators	Adhering leukocytes ↓ CXCL-2, IL-6 ↓	[95]
BEAS-2B cells	DFO	ROS	ROS generation ↓	[96]
Rats	DFO	TBARs	Lipid peroxidation ↓	[97]
Pigs	DFO	PLA2 activity	Alveolar collapse ↓	[98]
Rats	DFO	Oxidative stress	Oxidative damage ↓	[99]
Rats	2,3-DHB	Phospholipids	Peroxidation ↓	[100]
Sheep	2,3-DHB	Pulmonary response	Pulmonary arterial pressures ↓	[101]
Mice	DFX	Neutrophil activation	Neutrophil invasion ↓	[102]
Neutrophils	DFX	Neutrophils	ROS production ↓ NET formation ↓	[103]
Rats	21-aminosteroid	Elastin deposition DNA	Restructuring of newborn lung DNA injury ↓	[104] [105]

Note: CXCL-2, recombinant chemokine ligand 2; DFO, deferoxamine; DFP, deferiprone; DFX, deferasirox; DIBI, poly [(*N*-vinylpyrrolidone)-co-(3-hydroxy-1-[*N*-(methacrylamido) ethyl]-2-methylpyridin-4(1H)one; 2,3-DHB, 2,3-dihydroxybenzoic acid; iNOS, inducible nitric oxide synthase; NET, neutrophil extracellular trap; PLA2, phospholipase A2; ROS, reactive oxygen species; TBARs, thiobarbituric acid reactive substances. ↓= decrease.

## Data Availability

Not applicable.

## Abbreviations

ACSL4	Acyl-CoA synthetase long-chain family 4
AKI	Acute kidney injury
ALI	Acute lung injury
ARDS	Acute respiratory distress syndrome
CP	Ceruloplasmin
DcytB	Duodenal cytochrome B
DFO	Deferoxamine
DFP	Deferiprone
DFX	Deferasirox
DMT1	Divalent metal transporter 1
FPN	Ferroportin
GPX4	Glutathione peroxidase 4
HO-1	Heme oxygenase 1
IRE	Iron responsive elements
IRP	Iron regulatory protein
Lfn	Lactoferrin
LncRNAs	Long non-coding RNAs
LPS	Lipopolysaccharide
NET	Neutrophil extracellular trap
NGAL	Neutrophil gelatinase–associated lipocalin
NRAMP1	Natural resistance–associated macrophage protein 1
PLA2	Phospholipase A2
ROS	Reactive oxygen species
SARS-CoV-2	Severe acute respiratory syndrome coronavirus 2
TBARs	Thiobarbituric acid reactive substances
Tf	Transferrin
TfR	Transferrin receptor
YAP1	Yes-associated protein 1
ZIP8	Zinc transporter SLC39A8
